# Hereditary non-polyposis colorectal cancer: barriers to and facilitators of screening and disease management

**DOI:** 10.1186/1897-4287-9-S1-P39

**Published:** 2011-03-10

**Authors:** Kathy Watkins, Christine Way, Jacqueline Stokes, Robert Meadus, Mary Jane Esplen, Jane Green, Valerie Ludlow, Patrick Parfrey

**Affiliations:** 1 Centre for Nursing Studies, Eastern Regional Integrated Health Authority, St. John’s, NL, Canada; 2 School of Nursing, Memorial University of Newfoundland, St. John’s, NL, Canada; 3 Clinical Epidemiology Unit, Faculty of Medicine, Memorial University of Newfoundland, St. John’s, NL, Canada; 4 Department of Psychiatry, Faculty of Medicine, University of Toronto, Toronto, ON, Canada; 5 Department of Genetics, Faculty of Medicine, Memorial University of Newfoundland, St. John’s, NL, Canada

## Background

Hereditary non-polyposis colorectal cancer (HNPCC) is a hereditary cancer syndrome in which confirmed carriers of a gene mutation are at high risk for colorectal (CRC) and extracolonic cancers. The purpose of the current study was to develop a greater understanding of the factors influencing the decision-making of confirmed HNPCC carriers post-genetic testing about screening and disease management.

## Methods

The study used a grounded theory approach to data collection and analysis as part of a multiphase project examining the psychosocial and behavioral impact of genetic counselling and DNA testing for HNPCC on individuals in high risk families with the MSH2 intron 5 splice site mutation or exon 8 deletion. The data from confirmed carriers (n=23) were subjected to re-analysis for the purpose of identifying key barriers to and/or facilitators of effective screening and disease management.

## Results

Thematic analysis identified personal, health care provider and health care system as the dominant barriers to and facilitators of screening and disease management. Person-centered barriers/facilitators included (1) risk perceptions and decision-making and, (2) enduring screening/disease management. Provider barriers/facilitators were defined in terms of participant perceptions of physician awareness of the family history of HNPCC, knowledge of the disease and recommended screening/treatment protocols, and clinical management skills. The health care system barriers/facilitators were defined in terms of continuity of care and coordination of services among different providers.

## Conclusions

Individuals at high risk for HPNCC-related cancers often encounter multiple barriers to and facilitators of screening and disease management that go beyond the individual and family to the provider and health care system levels. The current organization and implementation of health care services for clients who are at high genetic risk of developing cancer is inadequate. A coordinated system of local services capable of providing integrated, efficient health care and follow-up, populated by health care providers with knowledge of inherited cancer, is necessary to maintain optimal health in these families.

